# Female Doctors

**Published:** 1881-05

**Authors:** 


### EDITORIAL, ETC.

*Female Doctors.*—According to a published statement the Women's Medical College of Philadelphia has turned out in its brief history one hundred and eighty graduates. All but thirty of these have put out signs. One hundred and fifty-one are yet in practice; and of those it is reported that correspondence with them by the dean elicits information from seventy-six, who have an average annual income of nearly $3,000, while four of the seventy-six enjoy incomes ranging from $15,000 to $20,000. Three only report that professional duties have prevented marriage, and forty-three state that their profession and marriage has been not only not incompatible but helpful and favorable to their relations as wives and mothers. Six think the good and evil effects of married life about balanced, and one only reports her profession as ill in its effects on her domestic married life.

As we have had in the past a few ladies (solely from Germany) in the classes of the Baltimore College of Dental Surgery, it would be of interest to know how many have retired from practice, how many find their work incompatible with domestic affairs, how many are succeeding well, &c. If this should meet the eye of any who can furnish such information, we will be glad of responses.

